# ADIIT — An Evidence-Based Maturity Model for Faculty Support Teams in the Health Professions

**DOI:** 10.1007/s40670-024-02229-z

**Published:** 2025-01-27

**Authors:** Dina Kurzweil, Karen Marcellas, Linda S. Macaulay

**Affiliations:** 1 Uniformed Services University of the Health Sciences, Education & Technology Innovation Support Office, 4301 Jones Bridge Rd., Bethesda, MD 20814 USA; 2 The Henry M. Jackson Foundation for the Advancement of Military Medicine, Inc., in support of the Uniformed Services University of the Health Sciences, Education & Technology Innovation Support Office, 4301 Jones Bridge Rd., Bethesda, MD 20814 USA

**Keywords:** Evidence-based practice, Faculty support, Health professions education, Maturity models, Change management

## Abstract

Faculty support teams in health professions education operate in a unique environment that can engender specific challenges in their operations and development. This paper introduces an evidence-based maturity model designed to support the establishment and growth of such teams. The ADIIT model, which is named for the five levels of maturity that such teams can attain: (1) Ad Hoc/Initial; (2) Defined; (3) Integrated; (4) Innovative; (5) Transformative, enables faculty support teams to assess their current state and identify areas for improvement. Key elements of performance included in the model are team, leadership, operations/focus, customer base, and auxiliary services. The paper describes criteria for each element at each level and provides guidance about how to advance to the next level. A fictional case study is provided to demonstrate the application of the maturity model. Using this model will help faculty support teams in health professions education to develop the high level of focus and professionalism necessary to navigate between and provide support for classroom education, distributed learning, and clinical education.

## Introduction and Background: The Need for the Model

The development of organizations that support faculty in their teaching — what we call *faculty support teams* — has been found beneficial in implementing evidence-based teaching practices, supporting educational scholarship, facilitating curriculum change, and breaking down silos [1–3]. The staff of such teams can include instructional and curricular designers, multimedia developers, graphics and media specialists, and evaluation and research experts. These teams can provide professional development opportunities as they assist faculty with enhancing their teaching skills and technology use, and can also support the institution in the accreditation process.

Faculty in health professions education have extensive backgrounds and specialties in the health sciences, but many of them have not had the opportunity to develop effective teaching skills as they have expanded their professional repertoire. As well, the type of teaching that is done in the health professions can require specific skill sets. For example, faculty may need to teach and assess students using simulations and manikins, or they may need to facilitate small group discussions. Faculty support teams can help them develop these skills as well as enhance their teaching in other ways [4].

Yet, there is often confusion about how to build these teams and what their growth should look like [1, 2]. *Maturity models* for faculty support teams in health professions can alleviate that confusion by providing a framework for planning, developing, and evaluating these teams in ways that improve their effectiveness and allow them to diversify and expand the support they provide to their institutions. In general, maturity models provide a framework that organizations can use to consider qualitative and quantitative information when examining people, culture, processes, structures, objects, and technology in order to assess the current situation and identify reasonable improvements over time. These models typically consist of a set of stages or levels that represent increasing levels of maturity, with each stage reflecting specific characteristics and capabilities [5]. Each stage or level entails a number of defined steps to provide a path for incremental and continued process improvement.

Classic maturity models were developed by organizations such as the Carnegie Mellon University Software Engineering Institute, IBM, Deloitte, and Gartner. Other maturity models, such as Information Technology Infrastructure Library and Information Technology Service Management, focus on the information technology (IT) management and service perspective. Beyond engineering and IT, there are maturity models for areas such as E-Learning, Online Leadership [6], Project Management [7], frameworks for knowledge [8, 9], and many more. Yet, a comprehensive literature review found no mention of maturity models specifically related to faculty support teams within medical/health professional schools. Even entities concerned with quality in areas relevant to health professions education, such as the Liaison Committee on Medical Education (LCME), International Society for Technology in Education (ISTE) [8], and the Project Management Institute (PMI), have general standards related to skills and processes needed for faculty support but no maturity models for faculty support teams. There are some maturity models for the creation of distributed/online learning [10–12], but those focus on one small element of what faculty support teams do; they are too limited in scope to guide the development of a team that provides comprehensive services. This paper alleviates that gap by presenting a maturity model specifically designed to encompass the multiple roles, skill sets, and capabilities required by a faculty support team in health professions education.

This paper lays out a novel five-level maturity model for faculty support teams in health professions education: the Ad Hoc/Initial-Defined-Integrated-Innovative-Transformative (ADIIT) maturity model. The proposed ADIIT maturity model, which can be used to help institutions as they create or further develop such teams, is based on the Capability Maturity Model (CMM) and its successor, the Capability Maturity Model Integration (CMMI), as well as standards from the LCME, ISTE, PMI, and others. This model does not eliminate other maturity models for instructional design, learning technologies, etc., but rather augments them in a way that gives precedence to the unique capabilities and circumstances of faculty support teams in health professions in higher education. It provides focus on the growth and maturity of such teams and describes the difference between immature and mature organizations. The paper defines the elements of the model, describing each element at each level in detail, and shows how to progress from level to level. It concludes with a case study and analysis to apply the maturity model to a fictitious health professions university.

## The ADIIT Maturity Model for Faculty Support Teams in Health Professions

Faculty support teams in health professions education operate in a unique environment that can engender specific challenges in their operations and development. They must be at home in the worlds of healthcare and academia, which requires a high level of focus and professionalism, as well as the ability to navigate between and provide support for classroom education, distributed learning, and clinical education. The ADIIT model centers on five main elements of faculty support teams that contribute to their success in such environments: the team itself, the leadership, the operations/focus of the organization, the customer base, and the types of academic advancement or auxiliary services provided by such teams.

- The **Team** must have skills that are aligned with the needs of the institution and must be willing to incorporate processes and structures that enable them to work efficiently in that environment.
- The **Leadership** must develop and maintain the team and manage the organization’s relationships with institutional leadership and other entities within the institution to set the team up for success, and must ensure that the team is connected with the scholarship of teaching and learning [13].
- The **Operations/Focus** of the team must be on evidence-based practice. The team must seek to develop relationships and trust that allow them both to collaborate with faculty on the design of instruction and to help the faculty develop the skills to implement instructional and technology innovations.
- Identifying and growing the **Customer Base** is an important element of the work of faculty support teams. Trust and relationships take time to grow, so the way the faculty support team interacts with customers and how their voice and ideas are heard changes over time. One way to start thinking about the evolution of the customer base as the organization develops is to integrate Rogers’ [14] *Diffusion of Innovation* adopter categories of Innovator, Early Adopter, Early Majority, Late Majority, and Laggards. Each group requires different types of support and can serve to focus initiatives and gain feedback. Using the Concerns Based Adoption Model from Hall and Hord [15] can help strengthen buy-in on the implementation of faculty support teams by intentionally taking into consideration the unique perspectives of all stakeholders and addressing their concerns.
- Providing proof of the team’s effectiveness and value to the institution can support the team’s development and also accelerate the growth of the team’s collaborative relationships with faculty. Incorporating the **Auxiliary Services** of professional development, research, and evaluation as part of their portfolio strengthens these efforts.

In using the ADIIT model, these five elements are assessed with respect to the level at which the team performs. There are five levels, which will be defined in the next sections of this paper: Ad Hoc, Defined, Integrated, Innovative, and Transformative.

**Fig. 1:**
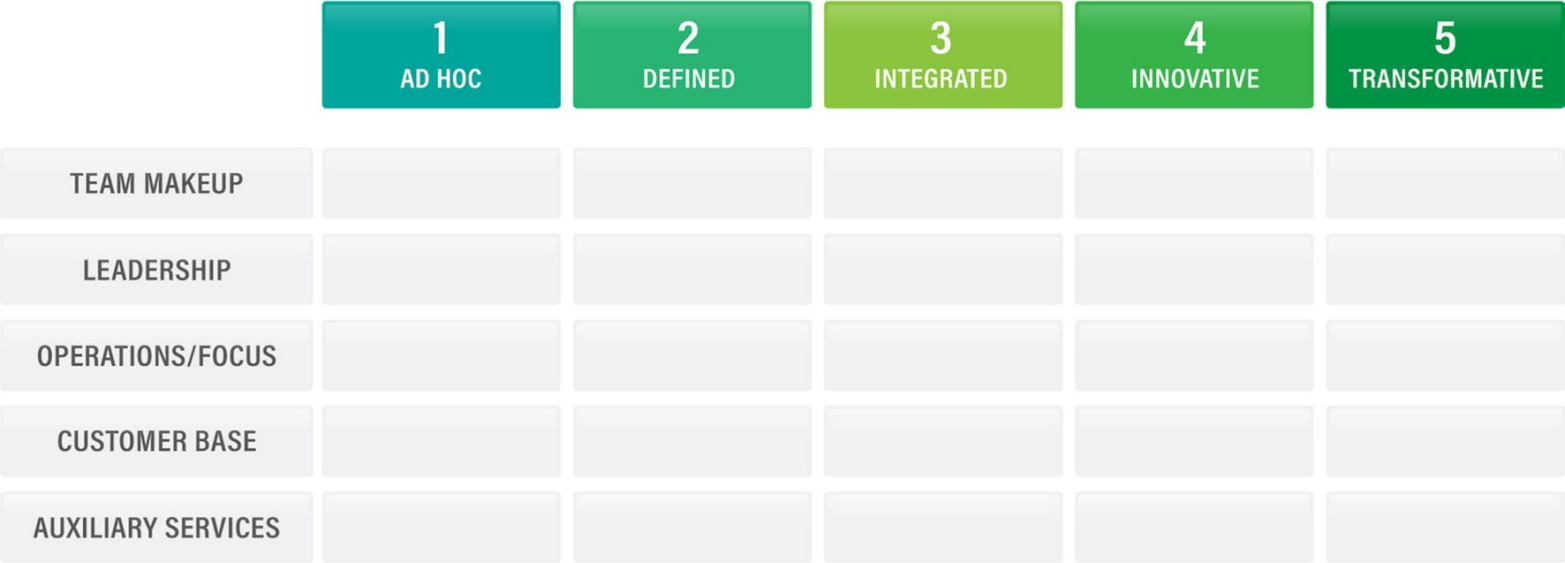
The ADIIT maturity model

Figure 1 depicts the levels and elements of the ADIIT model in a “dashboard” format that can display the maturity levels of the organization at a glance. It is possible for an organization to be at different levels for each of the elements and each element does not need to begin at level 1. The goal of the model is primarily to help teams identify how to get to the next level in the areas where it is possible for them to advance, not to just identify where the team lies on the ADIIT spectrum. To that end, for each level, the guidance provided for how to move to the next level incorporates recommendations for many different elements. Team leadership can then identify which steps are appropriate to help their teams mature in ways that are best suited to the team and the institution they support.

## Level 1: Ad Hoc/Initial

### Description

At this stage, the team may have limited resources and a lack of formal processes. Support is typically provided in an ad hoc manner; it is reactive and focuses on individual requests.

### Key Elements

- The Team — The team is small, with some specialties identified but no formal structure. Only limited types of work can be done since the team has limited skills. Roles within the team are loosely assigned.
- Leadership — There is no clear leadership or vision. Relationships with other faculty support organizations (e.g., library, simulation center) have not been established. The leadership may know of existing overlapping services at the institution but has not studied them systematically.
- Operations/Focus — Limited analysis on needs has been done to highlight specific support services to be provided. Activities and support are ad hoc, seen as a novelty, and uncoordinated within the ecosystem. Limited processes are in place for project management and guidance of work on projects. The faculty support model is based on the team doing work for the faculty; the faculty support team provides no customer (faculty) education on doing the work themselves nor moves to develop partnerships with faculty.
- Customer base — The team focuses on only a small subset of the available customer base. Most customers are early adopters [14] and innovators. Trust and relationships are established ad hoc.
- Auxiliary Services Provided
  - Professional Development — Activities are provided upon request.
  - Research — There is no clear vision or plan for collaborative research.
  - Evaluation — There is no evaluation or improvement process for the faculty support team nor a plan for providing data on institutional value.

### How to Move to Level 2

- The Team — Develop team expertise and structure.
- Leadership — Define leadership reporting structures. Review current competencies of the team and identify needed skills. Define needs and budget for both team and support and make plans for hiring and/or staff development. Ensure senior leadership is in place. Do a scan of the learning ecosystem to identify other organizations to partner with as well as develop a sense of the support team’s place in the institution’s ecosystem and hierarchy.
- Operations/Focus — Begin developing processes and documentation for project work as well as plans for incorporating faculty education into project work.
- Customer Base — Focus on early adopters approach and develop plans for raising awareness of available skills and services. Begin to foster/develop repeat customers. Start to internally and externally showcase results.
- Auxiliary Services Provided — Start the development of plans for providing professional development, research, and evaluation support.

## Level 2: Defined/Structured

### Description

The team begins to define itself and establish formal structures, processes, and resources to support faculty. Services become more consistent and standardized, and the team may provide basic training/professional development and resources for teaching and learning. Support is still mostly reactive, but begins to address more than individual requests.

### Key Elements

- The Team — The team has grown, with a clear structure, and has members with clearly defined skill sets and some specialties and subspecialties. More types of work are possible because the team has grown. Roles within the team are assigned.
- Leadership — There is a designated leader and a shared vision. Relationships with other faculty support organizations (e.g., library, simulation center) are in progress. Activities with and in support of them are in the early planning stages. Studies of overlapping services are completed and documentation about them is provided to the institution.
- Operations/Focus — Formal analysis of the need for support services is conducted. The team has begun to research and develop, follow, and document work processes. The faculty support team primarily does the work for the customer but incorporates an element of customer education to allow the customer to learn new tools and techniques.
- Customer base — The team focuses on only a small subset of the available customer base. Most customers are early adopters [14] but the early majority may begin to use services. Many customers are repeat customers but the team is beginning to try to market itself to bring new customers in. Trust and relationships are beginning to be established in a more formal manner.
- Auxiliary Services Provided
  - Professional Development — Activities are offered on an ad hoc basis, but limited in scope.
  - Research — Initial conversations about the office/department plans for research are in progress.
  - Evaluation — Development of a plan for collection of data on institutional value is beginning.

### How to Move to Level 3

- The Team — Continue to grow the team and ensure that there are clearly defined roles and specialties, building up instructional design and media services.
- Leadership — Continue to define needs and budget for both team and support. Begin developing relationships outside the organization’s learning ecosystem.
- Operations/Focus — Standardize use of work processes and documentation; increase attention to customer education during projects.
- Customer Base — Refine strategy and focus on early majority stakeholders. Build trust and relationships through repeat customers. Begin to develop a menu of services and plans for raising awareness of services.
- Auxiliary Services Provided — Plan regular professional development opportunities, begin thinking about research plans internally, and begin developing plans for evaluation.

## Level 3: Integrated/Optimized and Collaborative

### Description

In Level 3, the team becomes more integrated with the institution overall and collaborates and plans with other academic departments/units. The team develops partnerships and engages in cross-functional activities throughout the learning ecosystem to support educational development and services. Processes are optimized for long-term implementation. This may include proactive planning for needed pedagogical support, workshops, and/or technology integration support.

### Key Elements

- The Team — The team has the majority of roles filled and is working on identifying gaps in skills that can affect its ability to provide comprehensive support. It has a clear structure, but is not at operational capacity. Team members have clearly defined specialties and subspecialties. The type of work that can be done continues to expand. Roles within the team are assigned.
- Leadership — Leadership is involved in conversations at higher levels both within and outside the learning ecosystem. Relationships with other faculty support departments (e.g., library, simulation center) are in place, but with limited plans for support and collaboration. Leadership has reviewed documentation of overlapping services and begun to consider elimination or integration of such services.
- Operations/Focus — Team activities and support options are aligned with the needs analysis results; the team is beginning to gather data and metrics to determine effectiveness and efficiency. Standard processes and documentation are implemented and updated regularly. The team incorporates an element of customer education in projects, allowing the customer to learn new tools and techniques. Faculty are beginning to see the organization as a partner rather than a service provider.
- Customer base — Early majority start to actively use services [14]. New customers begin to make up a higher proportion of the customer base as small-scale efforts to raise awareness take hold. Trust and relationships are growing.
- Auxiliary Services Provided
  - Professional Development — Activities are planned to occur regularly but topics are determined on an ad hoc basis.
  - Research — Initial plans for internal research are in progress. There is no clear plan for collaborative research. The team has done presentations at conferences but not yet published.
  - Evaluation — Plan for gathering data on institutional value has been developed.

### How to Move to Level 4

- The Team — Work on filling identified personnel gaps. Focus on documentation and processes that allow cross-functional team members to cover for each other as needed.
- Leadership — Partner with stakeholders to prioritize core services and articulate future aspirations. Develop a budget for internal training. Continue developing relationships outside the organization’s ecosystem. Ensure that the faculty support team vision aligns with the institutional vision and provides institutional value.
- Operations/Focus — Refine and formalize standards and processes. Begin using data to inform operations and focus and begin to look towards institutional needs.
- Customer Base — Develop relationships with late majority customers and larger-scale efforts to raise awareness of services.
- Auxiliary Services Provided — Provide regular professional development opportunities in different modalities. Implement evaluation plans. Present and publish to further scholarship of teaching and learning at a national level.

## Level 4: Innovative Stage

### Description

The team adopts innovative practices and technologies to enhance faculty support. It leverages research and data to inform services and provide proactive and timely support; it actively contributes to the scholarship of teaching and learning. The team may also provide leadership and mentorship programs.

### Key Elements

- The Team — The team is fully developed, with a clear structure, and is at operational capacity. The type of work that can be done continues to expand. Roles within the team are assigned, but some team members are also cross-trained to take on additional roles as needed.
- Leadership — The leadership is seen as experts in the implementation of faculty support teams inside of the institution and is starting to make inroads nationally. Relationships with other faculty support departments (e.g., library, simulation center) are in place, with plans for support and collaboration as well as elimination or integration of overlapping services.
- Operations/Focus — The team is using data and metrics to become more effective and efficient. Standards and work processes continue to be refined as needed. The faculty support team does specific portions of the work based on expertise, with customer education that allows faculty to develop some or all skills needed to maintain the work/work products over time. Partnership relationships continue to develop. The group is a source of inspiration for activities and support to other groups within the ecosystem.
- Customer base — Late majority start to actively use services [14]. The proportion of new customers continues to grow, and the organization is using multiple channels to spread word about its skills and successes. Trust and relationships are established and expanding in the learning ecosystem.
- Auxiliary Services Provided
  - Professional Development — A specific program or series of professional development sessions is planned and offered, and sessions are also offered on an ad hoc basis as faculty needs arise.
  - Research — Team leverages internal research and data to inform services and actively contributes to the scholarship of teaching and learning through publishing and presenting at national conferences. Faculty projects are considered for research intentionally, and publications are planned in collaboration with faculty.
  - Evaluation — Plan for gathering data on institutional value has been disseminated. The group is a catalyst for positive change in the health professions education system within the institution. Institutional value reports are reviewed and used to evaluate for needs and change.

### How to Move to Level 5

- The Team — Continue to develop processes for cross functional coverage and operations.
- Leadership — Build active engagement both within and outside the ecosystem.
- Operations/Focus — Begin to focus on transformative and innovative practices that meet institutional needs.
- Customer Base — Focus on late majority and laggard [14] customers.
- Auxiliary Services Provided — Build a culture of quality and continuous improvements, use evaluation plans, and revise as needed. Present and publish internationally to further scholarship of teaching and learning.

## Level 5: Transformative Stage

### Description

At this highest stage of maturity, the team serves as a model of excellence and leads institutional initiatives related to faculty support. It engages in strategic planning, assessing and measuring its impact, and drives transformative change in teaching, learning, and faculty/staff development practices.

### Key Elements

- The Team — The team is fully developed, with a clear structure, and is at operational capacity. Roles within the team are assigned, but all team members are cross-trained to take on additional roles as needed.
- Leadership — The leadership is seen as experts in the implementation of faculty support teams and is leading conversations within and outside the institution internationally about faculty support, curriculum, and other areas of focus. The team is a leader in innovation and transformation. Relationships with other faculty support departments (e.g., library, simulation center) are in place and collaborative plans exist for joint projects. Overlapping services are eliminated and/or teams are integrated.
- Operations/Focus — The team has aligned its work to institutional needs, outcomes, and evaluation data, and may provide focused support to groups that are outside of their institution but have direct benefits to their institution. Standards and work processes are continuing to evolve. The faculty support model is based on educating the customer on new, transformative ways to meet both faculty and student needs, with the faculty and team members working in a collaborative partnership. The group is a source of support and inspiration for activities to other groups within the institution and has some limited outside influence.
- Customer base — Late majority start to actively use services, and laggards have just begun using services [14]. Trust and relationships evolve into new areas. The team has developed a reputation as a go-to organization for specific services.
- Auxiliary Services Provided
  - Professional Development — The team has a comprehensive portfolio of professional development, providing workshops and training opportunities as well as online offerings both independently and in partnership with other entities at the institution.
  - Research — The team is seen as actively contributing to the scholarship of teaching and learning with faculty, within the team, and with external groups. The team, frequently in partnership with faculty, publishes and presents at national and international conferences.
  - Evaluation — The group reviews plans for data collection of gaps, needs, value, and positive change in the learning ecosystem, and updates them regularly. Information gathered from reports is shared at national and international conferences or in publications.

## Case Study — University of the Clouds

This section of the paper provides a fictional example (drawn from the experiences of the authors at different institutions) of how an organization can use the ADIIT model to help identify its strengths as well as areas where growth will help it support health professions education even more effectively.

### The Faculty Support Team

The University of the Clouds has a small faculty support team, which has been in existence for 4 years, that includes:

- 1 Director (who has an instructional design background)
- 2 Instructional Designers
- 2 Graphics/Multimedia developers
- 1 Videographer

### ADIIT Key Elements

#### The Team

The team is not fully developed, but has a clear structure, and is able to meet the needs of most faculty at the university. The team engages in a variety of types of work, such as creating animations, assessments and assignments aligned to objectives, and professional learning videos, and interactive learning content. Team members have clear complementary roles but do not have overlapping skill sets. For example, one of the instructional designers has extensive experience with the university’s learning management system (LMS) and provides most of the team’s LMS support for faculty. There has been some discussion of training another instructional designer on the learning management system as backup.

#### Team Assessed Level: 3

##### Leadership

The Director is seen as an innovator and an expert in teaching and learning at the institution, leading conversations within the institution on faculty support and curricular planning, and participating actively in institutional committees and national groups. The Director is an advocate for transformation, helping to advance the institution’s policies and outcomes and take them in new directions. There are strong collegial relationships with the library and the simulation center, and collaboration is common on meaningful joint projects, such as professional development for faculty. The Director is involved in conversations about integrating into one team instructional design services that were previously spread throughout the university.

#### Leadership Assessed Level: 4

##### Operations/Focus

Data is sometimes gathered on effectiveness and efficiency via post-project feedback and student success data. Work processes are in place but have not been updated since they were created. The faculty support team incorporates some customer education when appropriate, allowing the customer to learn new tools and techniques as the partnership strengthens.

#### Operations/Focus Assessed Level: 3

##### Customer Base

There are many repeat customers, and the number of new customers is increasing. The team has created an intranet site that provides information about its services and is considering new ways to get the word out, such as an emailed newsletter, a blog, or an educational technology showcase. Trust and relationships are growing, and faculty support team members’ ideas are valued in conversations about the direction and specifics of projects.

#### Customer Base Assessed Level: 3

##### Auxiliary Services Provided

The team offers programmed professional development, providing workshops and training opportunities as well as online offerings both independently and in partnership with other entities at the institution. The team contributes to the scholarship of teaching and learning with faculty. The team presents at national and international conferences. The group reviews plans for data collection of gaps, needs, value, and positive change in the learning ecosystem and updates them regularly. Information gathered from reports is shared at national and international conferences or in publications.

#### Auxiliary Services Provided Assessed Level: 4

**Fig. 2:**
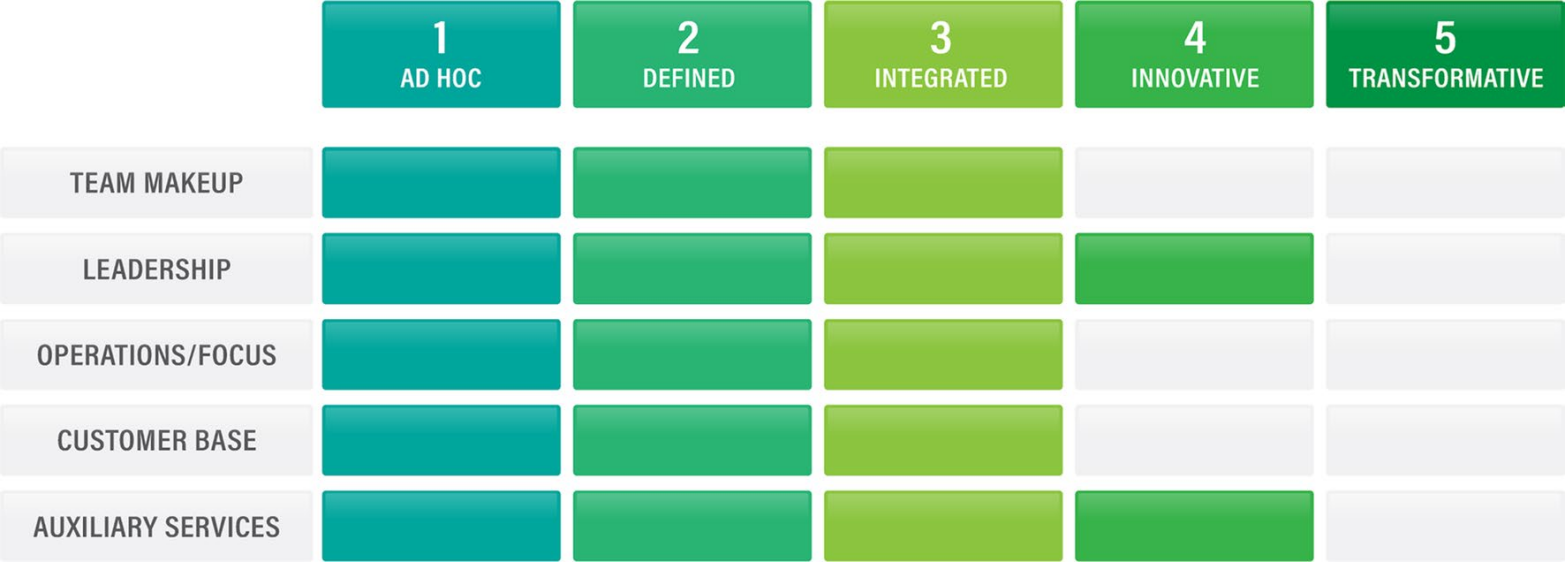
ADIIT maturity levels for the University of the Clouds (case study)

Figure 2 depicts the maturity for the University of the Clouds, as described in the case study.

### Analysis of University of the Clouds

The faculty support team has a number of pathways available to it that would help it increase its value to the health professions educators it supports:

- Team — Team members, in consultation with leadership, can pursue professional development opportunities that would enable them to develop skills in subspecialties, and could also cross-train in ways that allow them to back each other up in a variety of tasks.
- Leadership — The team still needs to hire an instructional designer with an evaluation/assessment background so that it can expand its service offerings and begin to be more involved with evaluation and research. Review of services overlapping with those provided by other support groups should result in increased efficiency through consolidation or removal of those services. The leadership still needs to build active engagement both within and outside the ecosystem through presentations and publications both nationally and internationally.
- Operations/Focus — The team should gather data about effectiveness and efficiency more consistently via post-project feedback and student success data in order to refine their processes and procedures. It should also review its work processes and procedures regularly to identify areas for evolution and improvement.
- Customer Base — While word of mouth seems to be helping the organization to build its customer base, the organization should be intentional about marketing its services in order to ensure it is able to reach as many interested faculty as possible, especially late majority [14] customers.
- Auxiliary Services Provided — In order to maintain a high quality of service, the team should build a culture of continuous improvement, use evaluation plans, and revise as needed. The team should also present and publish internationally in the realm of the scholarship of teaching and learning; involvement in conferences and publications will keep them abreast of advances and innovations in health professions education.

## Discussion: Setting the Team Up For Success Within the Institution

The ADIIT maturity model focuses attention on factors that help teams increase their value to health professions educators as they develop: team composition, collaboration, and the team’s relationship to other entities at the institution.

The composition and structure of the team and its leadership are crucial to the success of any faculty support team. These teams are often expected to be proficient in a wide range of skills that go beyond the actual design of instruction, including project management, building professional relationships, communication, graphics, interactive media creation, video editing, research, responding to shifting priorities, and promoting or defending their role to colleagues [2, 16].

As the team expands its skillset, making good hiring decisions contributes significantly to the strength of the team. Anderson et al. [1] found that hiring faculty support team members, specifically instructional designers, can be hard in health professions education, and helping leadership understand the importance of finding the right skilled candidate is difficult as well. Some of these hiring difficulties can be attributed to the unequal influences members of a learning ecosystem have over others, as opinion leaders will have the most influence in the learning community [14] but may not be experts in faculty support team development. It also can be hard because many people who make up faculty support teams are not seen as faculty members or as equal to faculty members but as support staff, exacerbating the unequal relationship [17, 18]. Ensuring that the team and its new hires have appropriate credentials can help the team gain credibility with faculty; team leadership should have at least a master’s degree and substantial work experience (ideally in the health professions education field). Likewise, as the team grows, leadership should ensure that it has an orientation process that allows new hires to work in tandem with experienced team members to build relationships with faculty.

Getting the right team in place enables the collaboration that is crucial to successful faculty support [1, 2, 19]. Two of the top qualities that enable a faculty support team to collaborate effectively with faculty and staff to improve and even transform student learning are authentic communication and trust [2, 20]. Both communication and trust may be viewed as necessary to being open to alternative ideas and thus transformation [2, 21, 22]. Growing faculty support teams into a mature stage involves developing relationships with faculty that allow for the faculty support team to be seen as a trusted partner with the faculty [2, 23] and develop structures to integrate and empower faculty support teams in high-level strategic initiatives [20]. In the realm of health professions education, faculty support teams can also enhance trust by ensuring that they take an evidence-based approach to designing and enhancing instruction, and that they make clear to faculty what the evidence is that supports the team’s approach and recommendations.

One barrier to the maturation of faculty support teams is that it can be hard to spread the word about a support group within a learning ecosystem. In order to increase use of an innovation like a faculty support team, Gustafson, Sanfort, Eichler, Adams [sic], Bisognano, and Steudel [24], found that intentionally “creating and communicating a mandate for change” (p. 753) about the innovation is likely to diffuse the innovation more quickly to the learning ecosystem. Increasing use of the faculty support can also be fostered via use of focused events, advertising, faculty champions, announcements at department meetings, and thorough onboarding. Such efforts at raising awareness can have additional dividends; Lee [25] revealed that faculty motivation, commitment, and satisfaction were even stronger as the faculty members felt they were well-supported by their schools.

An important consideration for both the team and leadership elements of faculty support teams is where the office/center will live within the learning ecosystem. According to Drysdale [17], there are two primary models for the placement of such teams within an institution’s organizational structure:

1. Centralized: one team serves all of an institution’s schools, colleges, and departments.
2. Decentralized: there are multiple teams, each of which is dedicated to one specific entity (school, college, and/or department).

Some institutions also may have a mix of decentralized and centralized placement, in which there are multiple teams and each team works for a specific entity, but the team reports to a centralized office/center (i.e., their role is placed in an organizational chart with the Dean, Provost, Vice President of Information Technology or Academic Vice President on the top).

Each of these two types of placement comes with their own advantages and disadvantages within the learning ecosystem. Some advantages of centralized models are a shared mental model, standardized practices, resource optimization, and reduction in duplication of effort. Some disadvantages of centralized models include limited team autonomy and the need to prioritize among a large number of organizations requiring services. For decentralized teams, one key advantage is the ability to develop trust and relationships faster. Disadvantages of a decentralized team involve the inconsistent implementation of standards, the creation of silos, and the possibility for friction within different teams providing faculty support. Faculty support teams can mature to the highest level under any of these models, but developing a clear understanding of the challenges and opportunities helps the organization develop intentional growth plans that are best suited to the environment. While it is easier to apply the ADIIT model to a centralized structure or a mixed structure, it can be applied to a decentralized structure if each team is seen as a separate entity that can mature at its own rate. However, the closer such a structure moves to the mixed model, the more they can take advantage of the efficiencies and standardized practices of the centralized structure, and thus gain cohesion by supporting each other’s growth. The report completed by Intentional Futures [20] helps us understand the challenges of these faculty support teams by highlighting how the organizational structure and leadership may act as significant barriers to growth and leadership, especially in the strategic planning phase, where leadership may not provide a seat at the table, or not listen to feedback from instructional designers. As well, the role of faculty support teams can also be very ambiguous, leading to stress if the role is misaligned with those with whom they work or others within the learning ecosystem [17, 26].

## Conclusion

Maturity models can help an organization grow over time [5]. The ADIIT Maturity Model for Health Professions faculty support teams help, institutions to create faculty support teams, evaluate growth, and plan the development of existing teams.

The ADIIT maturity model serves as a guide for health professions education faculty support teams, who can evolve from isolated or stand-alone units to valued members of the institution and the wider health professions education system. The model can be used to assess the current state of a faculty support team and to identify areas for improvement. It also acts as a roadmap for advancing and tracking services and capabilities over time.

## Declarations

### Disclaimer

The opinions expressed herein are those of the authors and are not necessarily representative of those of the government of the United States, the Uniformed Services University of the Health Sciences, the Department of Defense (DoD), or the Henry M. Jackson Foundation for the Advancement of Military Medicine, Inc.
